# Human Brainstem Exhibits higher Sensitivity and Specificity than Auditory-Related Cortex to Short-Term Phonetic Discrimination Learning

**DOI:** 10.1038/s41598-017-07426-y

**Published:** 2017-08-07

**Authors:** Stefan Elmer, Marcela Hausheer, Joëlle Albrecht, Jürg Kühnis

**Affiliations:** Auditory Research Group Zurich (ARGZ), Division Neuropsychology, Institute of Psychology, University of Zurich, Zurich, Switzerland

## Abstract

Phonetic discrimination learning is an active perceptual process that operates under the influence of cognitive control mechanisms by increasing the sensitivity of the auditory system to the trained stimulus attributes. It is assumed that the auditory cortex and the brainstem interact in order to refine how sounds are transcribed into neural codes. Here, we evaluated whether these two computational entities are prone to short-term functional changes, whether there is a chronological difference in malleability, and whether short-term training suffices to alter reciprocal interactions. We performed repeated cortical (i.e., mismatch negativity responses, MMN) and subcortical (i.e., frequency-following response, FFR) EEG measurements in two groups of participants who underwent one hour of phonetic discrimination training or were passively exposed to the same stimulus material. The training group showed a distinctive brainstem energy reduction in the trained frequency-range (i.e., first formant), whereas the passive group did not show any response modulation. Notably, brainstem signal change correlated with the behavioral improvement during training, this result indicating a close relationship between behavior and underlying brainstem physiology. Since we did not reveal group differences in MMN responses, results point to specific short-term brainstem changes that precede functional alterations in the auditory cortex.

## Introduction

The phonetic repertoire of a language is constituted by individual vowels and consonants as well as by the permissible combinations of these sounds^1^ to form consonant-vowel (CV) syllables. From a psychoacoustic perspective, a difference between the perception of consonants and vowels is that the former is rather dependent on the discrimination of temporal features like, for example, the voice-onset time (i.e., VOT)^2^, whereas vowel’s identity is more strongly mediated by frequency information (i.e., first and second formants, F1 and F2)^3^. The ability to meticulously discriminate spectral and temporal phonetic variations represents a ubiquitous prerequisite for the acquisition and establishment of a variety of language-related functions, including speech processing^4^, reading skills^5, 6^, and foreign language competence^7, 8^. Furthermore, it is noteworthy to mention that vowels are particularly interesting because they occur more often than consonants in any given language, and their repertoire varies quite strongly across languages. Consequently, an accurate perception and discrimination of vowels constitutes one of the most important phonetic constraints for learning a foreign language. The second argument that makes vowels particularly interesting is related to methodological approaches that enable to measure the neural responses to single formants in a highly specific manner^9, 10^. In this context, brainstem responses to harmonics sounds have previously been shown to encode the fundamental frequency (*f*0) and the formants of vowels with high fidelity, therefore providing a window into the specificity of brain changes as a function of learning^11, 12^.

During phonetic discrimination learning, acoustic information can be decoded at multiple levels along the auditory pathway, however with remarkable differences in terms of spectral and temporal resolution^10^. The brainstem and the auditory cortex constitute the two main computational entities of the auditory system, and exhibit a complex intertwining of reciprocal bottom-up and top-down projections that operate in a serial and parallel manner in order to refine how sounds are transcribed into neural codes^10, 13^. In this intertwining of processes, the auditory brainstem mimics the spectrotemporal characteristics of an auditory event with remarkable fidelity by interacting with the auditory cortex. The auditory cortex, in turn, integrates the incoming information, enables its transcription into higher cognitive representations, and subordinates the brainstem by providing direct modulatory influence via corticofugal top-down projections^14^. Currently, it is generally acknowledged that both the two main actors of the auditory system are highly prone to undergo functional changes^15–19^. However, until now only a few EEG studies have made use of test-training-retest procedures in order to track causal electrophysiological changes along the auditory pathway that emerge from short- (i.e., <1 hour) or long-term (i.e., >1 hour) phonetic discrimination training. At the cortical level, both short-^20, 21^ and long-term^22–24^ training protocols consisting in discriminating phonemes manipulated in formant transitions^20, 22, 23^ or syllables varying in VOT^21, 24^, have reliably been shown to induce causal changes in the auditory cortex, as reflected by a modification of auditory-evoked potentials’ (AEPs) strength. However, the physiological mechanisms underlying short- and long-term training are less clear. In fact, long-lasting training protocols have more often been associated with neural facilitation (i.e., increased AEP amplitudes)^22–24^, whereas short-term ones have been shown to induce both facilitation^20^ and adaptation (i.e., reduced amplitudes)^18, 20, 21^. The latter discrepancy possibly reflects different neural signatures of the acquisition and consolidation processes^25^. Finally, it is noteworthy to mention that the molecular, cellular, and physiological mechanisms beyond neural adaptation and facilitation as a function of training are not yet fully understood^26^. In fact, even though increased or reduced EEG responses have previously been related to the degree of synchronization of cell assemblies^27^, there are several other explanations that might account for these effects, including synaptic plasticity, short-term depression and facilitation, post-tetanic potentiation and hyperpolarization^26^, changes in neural tuning^28^, attention^29, 30^, as well as reward and motivation^31–33^.

To the best of our knowledge, until now only two EEG studies have examined causal functional changes at the processing level of the brainstem induced by phonetic discrimination training^11, 12^. Russo *et al*.^11^ as well as Song and colleagues^12^ made use of long-term training protocols consisting of learning to distinguish vowels manipulated in terms of pitch, and consistently revealed increased phase-locking to the fundamental frequency (i.e., *f0*) of the trained stimulus. Even though these results fundamentally contribute to a better understanding of the subcortical neural computations underlying phonetic discrimination learning, there are nevertheless several open questions that need to be addressed more deeply. First of all, it is unclear whether the human brainstem will show a more robust encoding after a single short-term training session. Second, it is still a matter of debate whether short-term functional changes in the brainstem will be manifested in terms of neural facilitation (i.e., increased amplitudes) or adaptation (i.e., decreased amplitudes)^28, 34^. Moreover, previous EEG studies focusing on vowel discrimination learning at the processing level of the brainstem^11, 12^ exclusively focused on pitch (i.e., *f*0 manipulations) and not on timbre (i.e., F1 and F2). Finally, it is important to mention that until now there are not so many studies that combined cortical and subcortical measurements^15, 35–37^ for attempting to describe putative cortical-subcortical coupling mechanisms between auditory cortex and brainstem while processing vowels or CV syllables, and none of them addressed training-related changes (for an overview also consider^38, 39^).

The present work aimed at contributing to a better understanding of the neural operations underlying short-term phonetic discrimination learning at both the processing level of the brainstem and the auditory cortex. In order to objectify signal-changes at the processing level of the brainstem we made use of FFRs, whereas modulations of the auditory cortex were assessed by means of MMN responses. The FFR is supposed to be generated by the inferior colliculus and the cochlear nucleus^13^, and has previously been shown to be synchronized to the periodicity of the sound with each cycle faithfully representing time-varying *f*0 and harmonics (i.e., f)^9, 10^. Furthermore, FFRs are highly replicable across test sessions and sensitive enough to capture subtle brain changes induced by training^11, 12^. By contrast, the MMN is an event-related potential (i.e., ERP) that is elicited by infrequent auditory events deviating in a specific physical dimension from a frequently presented standard stimulus^40^. Since the MMN is commonly elicited during passive listening paradigms, this ERP is supposed to be generated by an automatic stimulus-driven change detection process that is relatively unaffected by attention^41^. According to previous studies that combined EEG and fMRI measurements, the auditory-evoked MMN is mainly generated by the auditory cortex^42, 43^.

In the present work, we used EEG and performed repeated cortical and subcortical measurements (i.e., within the same day) in two groups of participants who underwent one hour of training consisting of discriminating CV syllables manipulated in F1 or were passively exposed to the same stimuli while watching a silent movie. The purpose of this study was to evaluate whether short-term speech discrimination training may suffice to induce a modulation of cortical and subcortical brain responses as well as to increase the functional interplay between the two computational entities. In addition, we re-evaluated the influence of short-term phonetic discrimination training on neural facilitation and adaptation.

## Results

### Psychometric and behavioral data

**Figure 1:**
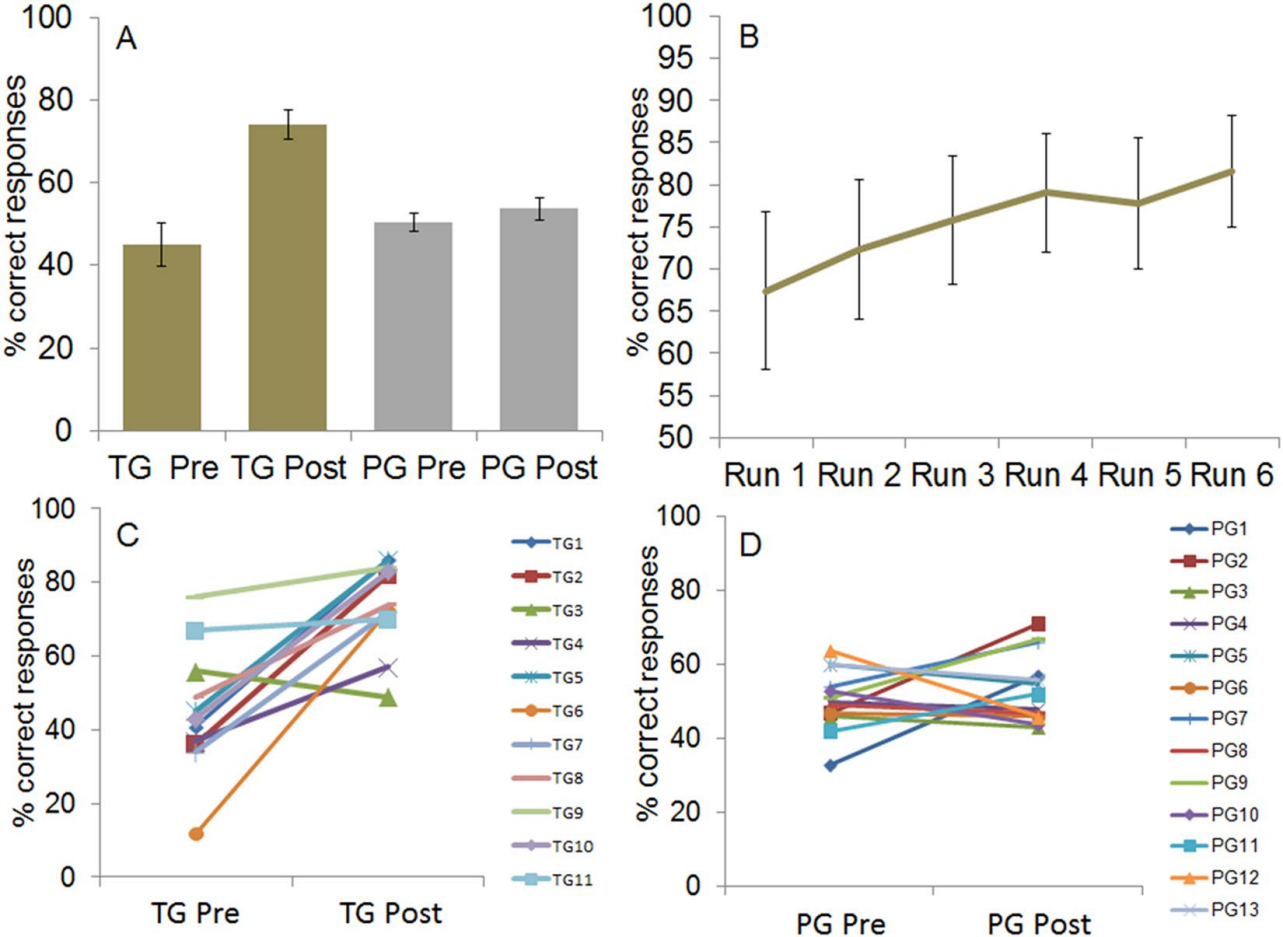
(**A**) Mean percent correct responses during the discrimination test at T0 (pre) and T1 (post) for the TG (gold bars) and the PG (silver bars). (**B**) Behavioral performance (mean percent correct responses) of the TG across 6 training runs. (**C**) Percent correct responses of the single participants of the TG in the discrimination test at T0 (pre) and T1 (post). (**D**) Percent correct responses of the single participants of the PG in the discrimination test at T0 (pre) and T1 (post). Error bars indicate standard error of the mean.

The two groups did not differ in terms of general cognitive capability (KAI t_(22)_ = 0.423, p = 0.676; MWT t_(22)_ = 0.642, p = 0.528) or alertness (t_(19)_ = 0.617, p = 0.545). Otherwise, the generalized linear mixed model (i.e., 2 groups × 2 time points) revealed a main effect of time point (z = −2.391, p = 0.0403) as well as group × time point interaction effect (z = 2.016, p = 0.0438). As visible in Fig. 1A, the main effect of time point originated from a better discrimination at T1 (mean correct responses = 63.04%) compared to T0 (mean correct responses = 48%), whereas the group × time point interaction was related to a higher performance of the TG compared to the PG at T1 (mean correct responses, TG T0 = 45.09%, PG T0 = 50.46%, TG T1 = 74.09%, PG T1 = 53.69%).

### FFRs responses

**Figure 2:**
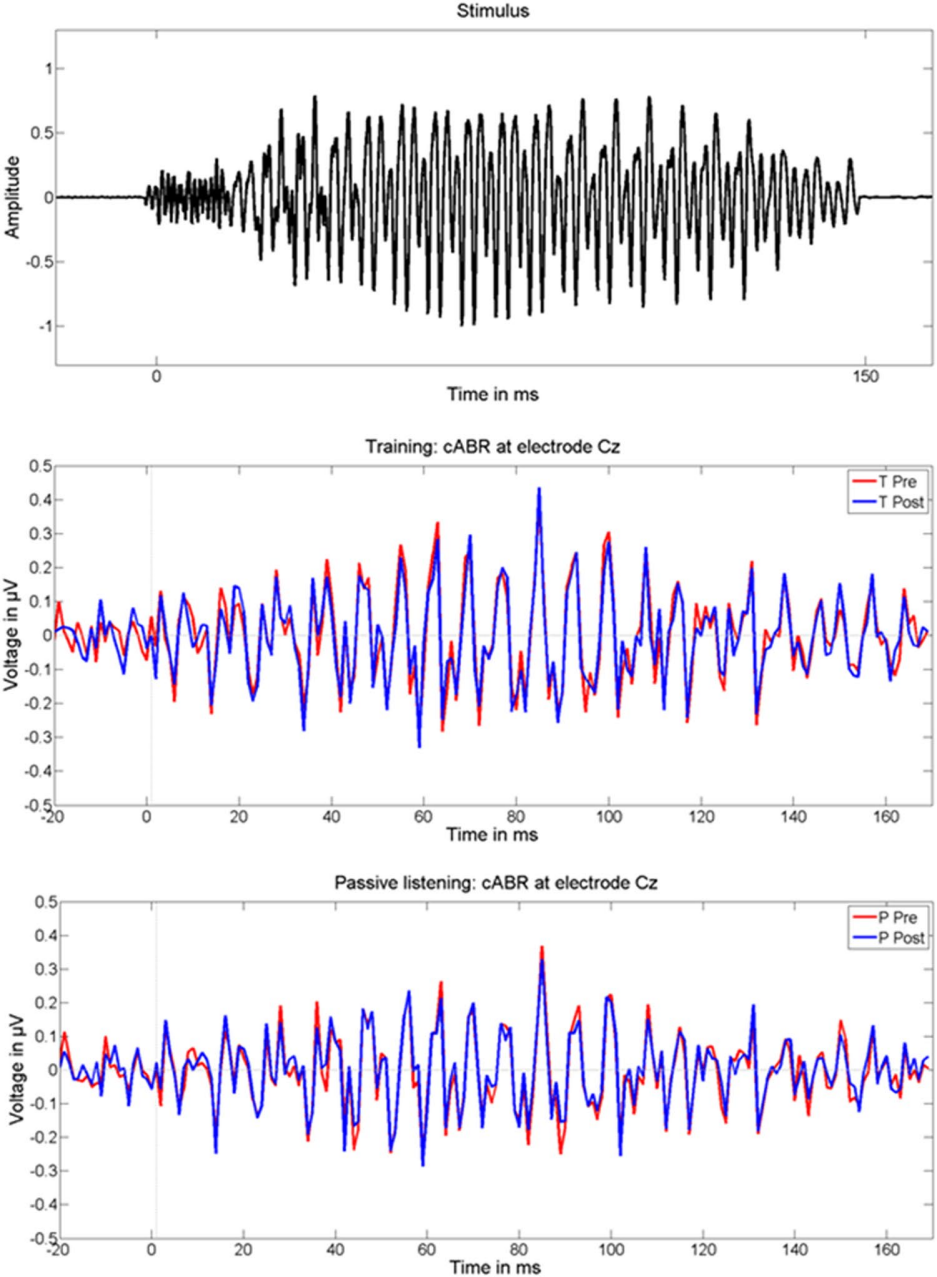
Stimulus waveform and FFRs (subtracted polarities). Top = stimulus waveform; middle = FFRs at T0 (pre, red line) and T1 (post, blue line) within the TG; bottom = FFRs at T0 (pre, red line) and T1 (post, blue line) within the PG.

A one-sample t-test computed against zero (i.e., no lag) across all participants in order to exclude electromagnetic interference induced by the headphones (Bonferroni corrected p value for two tests = 0.025) yielded significant results at both T0 (t_(23)_ = 7.825, p < 0.001) and T1 (t_(23)_ = 8.475, p < 0.001). These results are in line with previous literature^13^ and indicate the presence of genuine FFRs (Fig. 2) characterized by a mean delay of about 8 ms (i.e., T0, mean = 7.825 ms, sd = 1.917 ms; T1, mean = 8.475 ms, sd = 1.468 ms) reflecting signal transfer time from the ear to rostral brainstem structures^13^.

**Figure 3:**
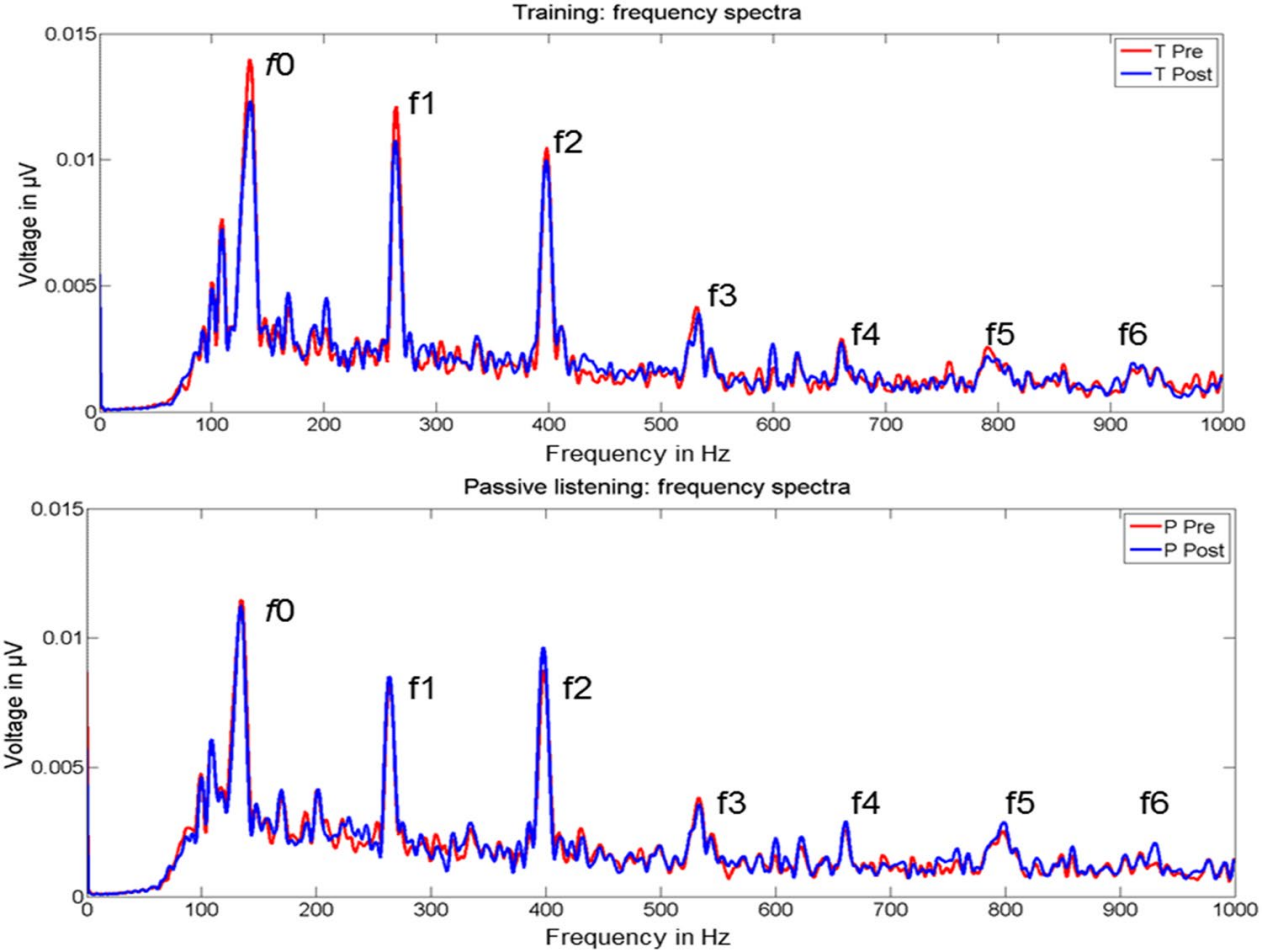
Grand-average FFR power spectra (i.e., subtracted polarities) at T0 (pre, red line) and T1 (post, blue line) of the TG (upper part) and PG (lower part). *f*0 = fundamental frequency, f1–f6 = harmonics.

**Figure 4:**
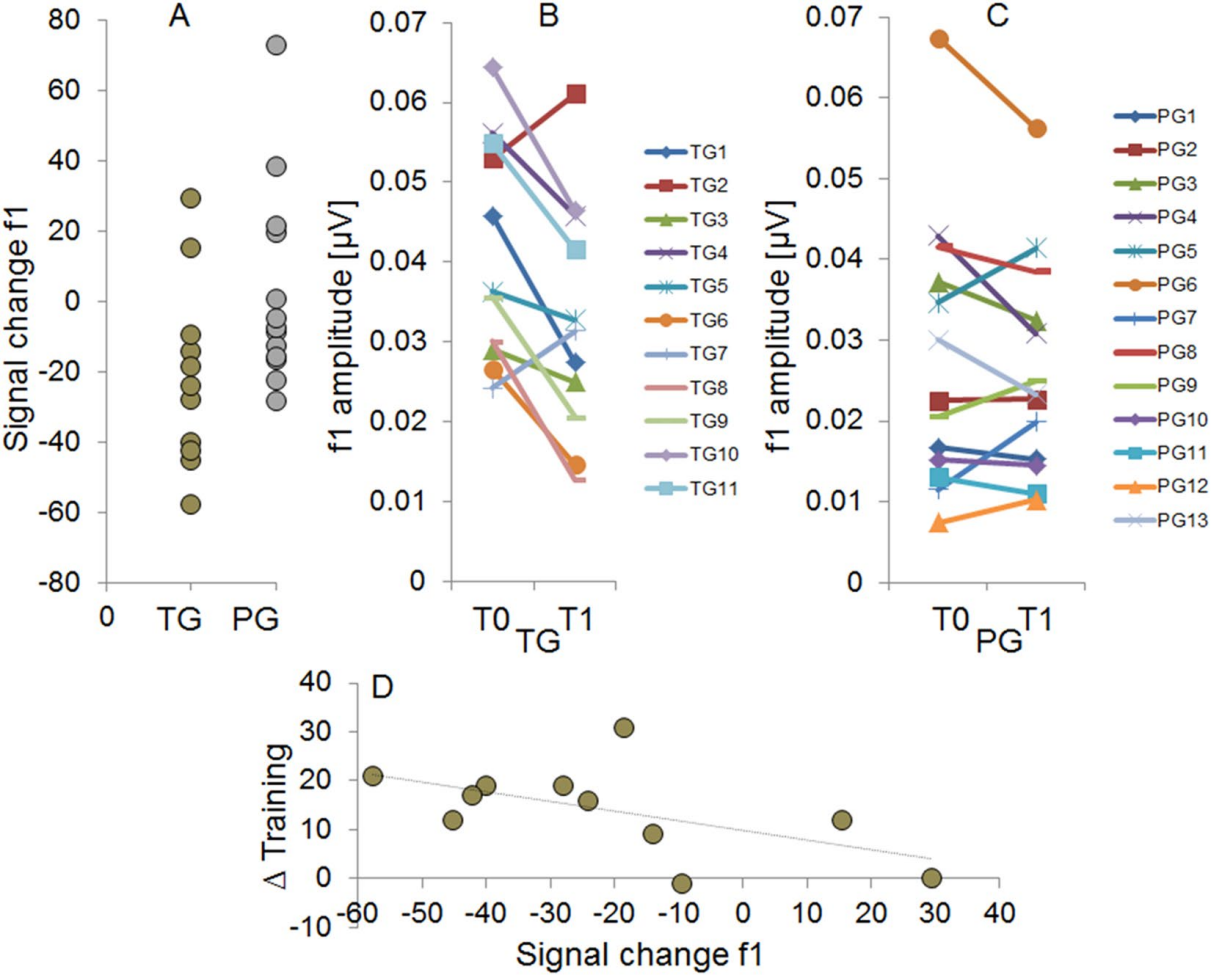
(**A**) Percent f1 signal change for each subject of the TG (gold) and of the PG (silver). (**B**) f1 amplitudes at T0 (pre) and T1 (post) of each participant of the TG. (**C**) f1 amplitudes at T0 (pre) and T1 (post) of each participant of the PG. (**D**) Significant correlation within the TG between percent f1 signal change and learning performance (Δ Training = percent correct responses run 6 minus run 1).

The evaluation of between-group f1 peak amplitudes (Fig. 3) by means of a t-test (i.e., percent signal change) yielded a significant group difference (t_(22)_ = −2.147, p = 0.043). Post-hoc t-tests against zero calculated separately for the two groups (i.e., Bonferroni corrected p value for two tests = 0.025) revealed that the TG was characterized by a significant signal reduction (t_(10)_ = −2.704, p = 0.022; mean % signal change = −21.36, neural adaptation, Fig. 4), whereas brain activity did not change within the PG (t_(12)_ = 0.349, p = 0.733, mean % signal change = 2.74). Finally, even though we did not have any a priori-hypotheses, for reasons of completeness, we also evaluated percent signal change in *f*0 (i.e., added responses) and higher harmonics (i.e., subtracted responses, f2, f3, and f4) between the two groups. Since we did not reveal group differences in these additional parameters (*f*0, t_(22)_ = −0.193, p = 0.849; f2, t_(22)_ = −0.881, p = 0.388; f3, t_(22)_ = −0.586, p = 0.564; f4, t_(22)_ = −0.035, p = 0.972), results indicate a specificity of brainstem responses to the trained stimulus attribute (i.e., f1).

### FFR: stimulus-response correlations

Potential group differences in stimulus-response correlations (i.e., stimulus tracking and lag) as a function of treatment were evaluated by contrasting the percent signal change between the two groups by means of t-tests (Bonferroni corrected p value for two tests = 0.025). These analyses did not reveal significant group differences in signal tracking (t_(22)_ = 0.508, p = 0.617) nor in lag (t_(22)_ = −0.182, p = 0.857).

### FFR: brain-behavior relationships

In order to provide further evidence for the specificity of the functional changes observed within the TG at the processing level of the brainstem, we correlated percent f1 signal change with the learning performance during the training session (i.e., Δ percent correct responses between run 6 and run 1 of the training session). Results revealed a significant negative correlation (i.e., see Fig. 4D) between the two variables (r = −0.607, p = 0.024, one-tailed).

### MMN responses

**Figure 5:**
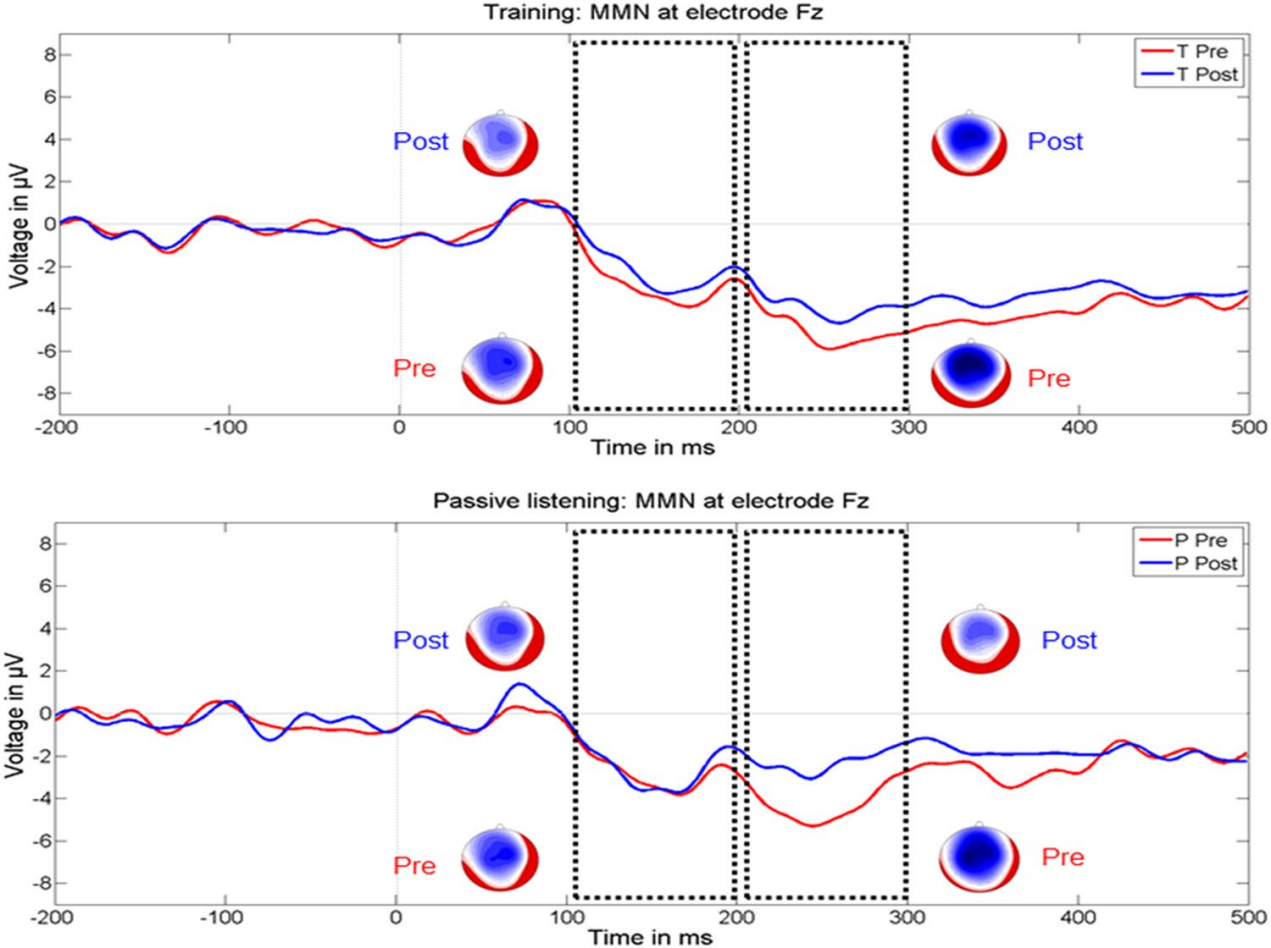
Grand-average MMN responses to spectral (early) and temporal (late) manipulations within the TG (upper part) and the PG (lower part) at T0 (pre, red line) and T1 (post, blue line). The black boxes indicate the time windows of analyses for both the early (100–200 ms) and the late (200–300 ms) MMN with the respective topographies. All waveforms are depicted at electrode FCz.

Between-group differences in MMN area and latency in response to spectral (i.e., early MMN) and temporal (i.e., late MMN) manipulations were evaluated by means of separate t-tests (i.e., percent signal change; Bonferroni corrected p value for 4 tests = 0.0125). These statistical analyses did not reveal significant group differences (spectral area t_(22)_ = −1.167, p = 0.256; temporal area t_(22)_ = 1.656, p = 0.112; spectral latency t_(22)_ = 1.085, p = 0.29; temporal latency t_(22)_ = −0.514, p = 0.613). Furthermore, in order to rule out the possibility that a general adaptation of the auditory cortex (i.e., see Fig. 5) as a consequence of repeated auditory stimulation between the two measurements points (i.e., T0 and T1) may have accounted for the lack of group differences, we performed additional post-hoc analyses within the two groups (one sample t-test against zero, two-tailed, Bonferroni corrected p value for 4 tests = 0.0125). These supplementary analyses did not reach significance (TG MMN area early, t_(10)_ = −1.536, p = 0.155; TG MMN area late, t_(10)_ = −1.493, p = 0.166; PG MMN area early, t_(12)_ = 1.173, p = 0.264; PG MMN area late, t_(12)_ = −2.466, p = 0.030).

### MMN sources

LORETA source estimation (Table 1) consistently revealed MMN maxima originating from posterior superior temporal areas, irrespective of group affiliation (i.e., TG and PG), time point (i.e., T0 and T1), and condition (i.e., spectral and temporal). These findings point to a main contribution of the auditory cortex to MMN responses.

**Table 1:** Reconstructed source maxima of the MMN responses in the time windows between 100-200 (spectral manipulation, early MMN) and 200-300 ms (temporal manipulation, late MMN) for the two groups (TG and PG) and the two time points (pre = T0 and post = T1).

Group	Early MMN		Late MMN		Talairach Coordinates	Brodmann Area
	Pre	post	Pre	Post		
TG	×				X = −59, Y = −32, Z = 8	BA 42
TG			×		X = −59, Y = −32, Z = 15	BA 42
TG		×			X = −59, Y = −32, Z = 8	BA 42
TG				×	X = −59, Y = −32, Z = 15	BA 42
PG	×				X = −59, Y = −32, Z = 15	BA 42
PG			×		X = −59, Y = −32, Z = 15	BA 42
PG		×			X = −59, Y = −32, Z = 15	BA 42
PG				×	X = −59, Y = −25, Z = 15	BA 40

.

### Training-related cortical-subcortical relationships

Putative changes in cortical-subcortical interactions within the TG were evaluated by correlating (according to Pearson’s r, two-tailed) the percent signal change of early MMN area and latency with f1 signal change (Bonferroni corrected p value for two tests = 0.025). These correlative analyses did not reach significance (r_MMN area_f1 amplitude_ = 0.155, p = 0.65; r_MMN latency_f1 amplitude_ = 0.355, p = 0.285).

## Discussion

### General discussion

In the present work, we used a test-training-retest procedure in two groups of participants who performed one hour of phonetic discrimination training, or were passively exposed to the same stimulus material, with the aim to (1) infer putative changes in the brainstem and auditory cortex as a function of short-term training, (2) estimate whether these short-term changes are reflected in neural facilitation or adaptation, (3) and to describe mutual interdependences between auditory cortex and brainstem. Results demonstrated that the brainstem but not the auditory cortex distinctively altered its response properties after short-term training. Most notably, this functional change was manifested in terms of neural adaptation and restricted to the frequency range (i.e., f1) corresponding to the trained stimulus attribute (i.e., F1). Since this frequency-specific neural adaptation was negatively correlated with the behavioral improvement of the participants during training, results point to a close relationship (~36% explained variance) between behavior and the underlying brainstem physiology.

### Brainstem responses

Nowadays, it is generally acknowledged that the human brainstem constitutes a highly plastic entity^13^ that can alter its response properties as a function of both long-^8^ and short-term training^34^. For example, Carcagno and Plack^44^ evaluated the FFR before and after ten hours of pitch discrimination training consisting of differentiating complex tones with a static-, raising-, or falling pitch contour, and found a more robust phase locking of the FFR to the static and dynamic *f*0 after training. Furthermore, neural activity in the brainstem has previously been shown to be specifically modulated as a function of long-term language experience as reflected by increased *f*0 magnitudes in Chinese compared to English speakers in response to iterated rippled noise with Mandarin pitch contours^45^ or high rising Mandarin lexical tones^46^. However, until now, only two EEG studies specifically addressed causal changes in the brainstem induced by speech discrimination training^11, 12^. In a first study, Russo and co-workers^11^ reported that after long-term training (i.e., 35–40 sessions of one hour each) children suffering from learning disabilities exhibited brainstem responses that were more resistant to the detrimental effect of background noise than before treatment. Similarly, Song and colleagues^12^ demonstrated that native English-speaking participants who learned to incorporate foreign lexical pitch patterns varying in *f*0 (i.e., 8 sessions à 30 minutes, accomplished in 14 consecutive days) were characterized by a more faithful representation of *f*0 stimulus contour.

In the present work, we provide evidence for short-term changes in the human brainstem after only one hour of phonetic discrimination training. However, contrary to previous studies that used professional musicians as a model for long-term training^8, 47^, results revealed functional changes that were manifested in terms of neural adaptation and not facilitation. Interestingly, a similar neural adaptation at the processing level of the brainstem has previously been reported by Slabu and colleagues^48^ in the context of a passive oddball paradigm. Thereby, the authors revealed a reduction of FFRs to deviant stimuli compared to standard ones, leading to suggest that the brainstem is able to encode statistical regularities^34^ by suppressing responses to rare stimulus events. Even though in the present study the “deviant” stimulus (i.e.,/go/) presented during brainstem measurements occurred with a low probability during the training session, the experimental manipulation we used precludes that results were driven by stimulus statistics^34^ or even by repetition suppression^28^. In fact, the PG was passively exposed to the same stimulus material as the TG, however, without showing a modulation of brainstem responses in pre-post comparisons. In addition, since brainstem changes were restricted to the solely discriminative physical attribute enabling to distinguish the trained stimuli, namely F1, results clearly point to feature-specific changes possibly reflecting increased neural efficiency^28^. This perspective is further supported by the negative correlation we revealed within the TG between percent f1 signal change and behavioral improvement during the training session.

Neural adaptation constitutes an intrinsic organizational property of the auditory system across the entire hierarchical tree, ranging from the periphery to the auditory cortex (for a review consider^49^). In this context, it is noteworthy to mention a previous EEG study targeting at evaluating the encoding of statistical regularities while participants learned to segment complex tone patterns embedded in concatenated sound sequences. Interestingly, the authors revealed decreased brainstem responses to the patterned compared- to a pseudo-random condition after only fifteen minutes of task^34^. However, by looking at brain responses of the single participants, Skoe and colleagues^34^ noticed that neural adaptation and facilitation can go hand in hand with remarkable inter-individual differences. Furthermore, the authors revealed a positive relationship between brainstem physiology and behavior, such that better performance was related to greater neural enhancement. Notably, our results are comparable with those of Skoe and colleagues^34^ in that the TG demonstrated decreased f1 magnitudes after short-term learning compared to the PG. Otherwise, in contrast to Skoe and co-workers, we revealed a negative instead of a positive relationship between the magnitude of brainstem responses and behavioral improvement. From a physiological perspective, the adaptation we revealed at the processing level of the brainstem can be explained at least by three different phenomena. The first possibility is that short-term training may have altered the response properties of brainstem neurons by uncoupling neural entities that were not relevant for discriminating task-specific acoustic features, resulting in activation of fewer neurons, and consequently neural adaptation^28^. A second possibility is that the observed brainstem changes may have been indirectly mediated by performance feedback. In fact, since in the present study only the TG received such a feedback, it is thoroughly possible that reward and motivation may have modulated brainstem activity. This perspective is supported by previous work showing that the human reward system is responsive to high-order rewards (i.e., intellectual, artistic, or altruistic pleasures)^31^ and that feedback confirming reward expectation can modulate activity in auditory-related brain regions^32, 33^. Finally, since active learning requires a stronger engagement of attention functions compared to passive listening, we cannot rule out that this variable may have played a role in mediating neural adaptation^29, 30^. Such an influence of attention could, for example, have been mediated by the cortex through corticofugal projections. In fact, such a contribution of the cortex to auditory learning mechanisms via the corticofugal system has previously been demonstrated in animals by using both ablation and pharmacological interventions^49, 50^.

A disadvantage of the EEG technique is that it does not enable to exactly determine the specific origin of the brainstem signal measured. However, currently there is evidence showing that neurons situated in the inferior colliculi are highly frequency-selective^51, 52^ as well as sensitive to the direction of frequency modulation^53, 54^. Since the TG was specifically trained to recognize subtle F1 signal changes only in one direction (i.e., always from/gu/to/go/, in the range between 364–480 Hz), we may speculate whether this specific experimental manipulation may have altered the response properties of neurons being selective to the direction of frequency modulation or rather frequency-selective neurons per se. In addition, since we did not reveal group differences in stimulus-response cross-correlations (i.e., lag and signal tracking), results suggest that during short-term training the brainstem is more likely prone to change its response properties to the spectrum of the trained stimulus attribute than to the waveform periodicity. This result is somehow in opposition with those previously reported by Russo and colleagues^11^ who revealed an increased temporal alignment of FFRs after training, as reflected by increased quiet-to-noise inter-response correlations. However, in this previous work the authors measured children with learning disabilities that were trained for a much longer period of time (namely 35–40 hours) compared to the present work. The same is true for the work of Anderson and colleagues^55^ where the authors evaluated the impact of an 8 weeks computer-based auditory training program in elderly subjects, and reported earlier brainstem peak latencies in both quiet and noise conditions after treatment. Taken together, these previous results substantiate the suspicion that brainstem changes in timing parameters may necessitate longer training periods.

### MMN responses

A further goal of this study was to evaluate the functional malleability of the auditory cortex as indexed by altered MMN responses. In addition, based on previous studies indicating that neuronal entities which are sensitive to temporal and spectral acoustic attributes lie side by side in the auditory cortex^56^, we evaluated putative transfer effects^57^ from phonetic discrimination training to temporal aspects of speech processing. Reconstructed sources revealed MMN maxima originating from posterior superior temporal areas across groups (i.e., TG and PG), conditions (i.e., spectral and temporal manipulation), and time points (i.e., T0 and T1). This finding is in line with previous literature^58^ and points to a main contribution of the auditory cortex to MMN responses. In the present work, we did not reveal group differences in the modulation of MMN responses (i.e., MMN area and latency) as a function of treatment, leading to suggest that the auditory cortex was not specifically modulated by training. Interestingly, previous training studies consistently revealed increased MMN responses that were accompanied by an improved behavioral performance, however, especially after multiple training sessions lasting several days or weeks^22, 59, 60^. In particular, Ylinen *et al*.^60^ measured native Finnish (i.e., quantitative language) and English speakers before and after 10 training session of 25 minutes each consisting of learning to discriminate spectral and temporal cues of English vowels. As a main result the authors reported that after training the Finnish speakers were better able to discriminate spectral vowel cues, as reflected by increased MMN responses. In a further EEG study, Tamminen and colleagues^59^ applied a three-day phonetic-listen- and repeat training in a sample of Finnish speakers who learned voicing contrasts in fricative sounds (i.e., fricatives are not differentiated by voicing in Finnish) and revealed significantly increased MMN responses after the second but not the first training day. Taken together, these previous results lead to suggest that functional changes in the auditory cortex can most reliably be induced by multiple training sessions. Therefore, we may speculate whether a consolidation period is necessarily required for inducing detectable plastic changes in the auditory cortex^61, 62^.

An alternative explanation that may account for the apparent insensitivity of MMN responses to training is that the constant serial order of the cortical and subcortical EEG measurements (i.e., FFRs were always collected first) may possibly have blurred neural facilitation through a superimposed signal adaptation. However, since between the two measurement points (i.e., T0 and T1) the two groups were additionally exposed to acoustic stimulation for one hour, we should have observed such an effect in pre-post comparisons (i.e., a significant percent MMN change against zero), irrespective of group affiliation. Finally, based on the fact that phonetic discrimination learning is an active perceptual process that operates under the influence of attentive functions, future training studies should evaluate short-term changes in the auditory cortex by combining active and passive oddball paradigms.

### Cortical-subcortical coupling mechanisms

To the best of our knowledge, until now only four studies conjointly recorded FFRs and AEPs while participants were exposed to CV syllables^36, 37^ or vowels^15, 35^. In particular, Musacchia and colleagues^36^ measured musicians and non-musicians while participants were repeatedly exposed to the syllable/da/, and reported a positive relationship between subcortical *f*0 amplitude and cortical P1-to-N1 slope. Otherwise, Bidelman and colleagues^35^ measured young and older adults while the participants categorized vowels that spanned a perceptual continuum from/u/to/a/and revealed that older adults were characterized by slower and more variable speech classification performance than younger listeners. This differential behavioral performance was reflected by reduced brainstem amplitudes, increased cortical AEPs, as well as by a negative relationship between f1 and cortical N1/P2 amplitudes. In a second study of the same group^15^, the authors recorded cortical and subcortical brain responses in older adults with and without music training while the participants categorized vowels along a continuum. Even though the authors did not find between-group differences in terms of cortical (i.e., P1-N1-P2 complex) or subcortical (i.e., *f*0 amplitude) brain responses, musicians showed a closer relationship between neural activity and behavioral performance. Finally, Parbery-Clark *et al*.^37^ investigated the effect of background noise on both brainstem and auditory cortex activity, and reported a relationship between subcortical response fidelity and cortical N1 magnitude that was predictive of speech-in-noise perception. In the present work, we did not find evidence for a relationship between auditory cortex and brainstem changes as a function of training. However, this may rather be a byproduct of unmodulated MMN responses as a function of training rather than an evidence for the inexistence of cortical-subcortical coupling mechanisms. In this context, it is also important to mention that our experimental design profoundly differed from the previous studies mentioned above. In fact, Musacchia and colleagues^36^ as well as Bidelman *et al*.^15^ measured musicians, a specific group of subjects that has previously repeatedly been shown to constitute a suitable model for evaluating the influence of long-term training on auditory processing^16, 63, 64^. Otherwise, the group of Parbery-Clark^37^ evaluated cortical-subcortical coupling mechanisms in normal hearing young adults while performing a speech-in-noise perception task, an experimental condition which is well known to place stronger demands on cognitive control mechanisms that have a modulatory influence on brainstem activity through the corticofugal system^34^.

## Conclusions

In summary, our results highlight causal and feature-specific changes in the human brainstem after only one hour of phonetic discrimination training, as reflected by neural adaptation in the frequency-range (i.e., f1) corresponding to the trained acoustic feature (i.e., F1). Since these brainstem changes correlated with the behavioral improvement of the participants during the training session, results are interpreted as reflecting neural efficiency induced by short-term phonetic discrimination training.

## Materials and Methods

### Participants

We evaluated the EEG data of two groups of subjects who were repeatedly measured within the same day (i.e., at time point 0 (T0, pre) and time point 1 (T1, post)) by using EEG protocols that enable the collection of both cortical and subcortical brain responses. Between the two measurement points, one group underwent active phonetic discrimination training (i.e., training group, TG, 11 subjects, 3 men, mean age = 23.54 years, SD = 3.04 years), whereas the second one was passively exposed to the same stimulus material while watching a silent movie (passive group, PG, 13 subjects, 3 men, mean age = 23.84 years, SD = 2.44 years). All participants were in the age range of 20–30 years, of German mother tongue, non-bilinguals (i.e., did not grow up with more than one language before school), non-musicians, and consistently right-handed^65^. None of the participants reported a history of neurological, psychiatric or audiological disorders. In addition, all participants were tested with pure-tone audiometry (MAICO Diagnostic GmbH, Berlin) in the frequency-range of 250–8000 Hz (MAICO Diagnostic GmbH, Berlin). According to this procedure, all participants demonstrated an unremarkable audiological status (i.e., all tested frequencies could be heard below a threshold of 30 dB). The participants were paid for participation, the local ethics committee (i.e., Kantonale Ethikkommission Zurich) approved the study (in accordance with the Helsinki declaration), and written informed consent was obtained from all participants.

### Cognitive capability

In order to test for group differences in cognitive capability, each participant performed two German intelligence tests, namely the MWT-B and the KAI (MWT-B, Mehrfachwahl-Wortschatz Intelligenz Test; KAI, Kurztest für allgemeine Basisgrössen der Informationsverarbeitung). The MWT estimates crystallized intelligence, and has previously been shown to correlate fairly well (r = 0.72) with the global intelligence quotient in healthy adults^66^. This specific test consists of 37 items which are ordered as a function of difficulty level. For each item, the participants have to choose the unique word with a meaning out of five pseudo-words. By contrast, the KAI estimates fluid intelligence, and is based on short-term memory (i.e., number- and digit span forward) and speed of information processing (i.e., reading aloud rows of random letters as fast as possible). Finally, tonic arousal, a variable which is known to have an influence on learning mechanisms, was assessed by using a subtest of the TAP test battery (Testbatterie zur Aufmerksamkeitsprüfung). During this test, participants were instructed to react as fast as possible whenever a white cross appeared randomly on a black screen. Due to a bug in the software, three participants (i.e., one of the TG and two of the PG) could not be tested on this task.

### Stimulus material

The auditory stimuli consisted of two semi-artificial German CV syllables, namely/gu/and/go/, which were created using PRAAT. The original syllable/go/was spoken by a male speaker, and recorded at a sampling rate of 44.1 kHz. In a first processing step, the consonant/g/was separated from the vowel/o/by identifying the time period between consonant burst onset and the onset of periodic oscillation taken to indicate vocal fold vibration. Afterwards, the vowel was replaced by fully artificial ones with identical fundamental frequency (i.e., *f*0 = 130 Hz) and second formant (i.e., F2 = 860 Hz) but different first formant (i.e., F1) (i.e., F1/u/ = 364 Hz, F1/o/ = 480 Hz). The artificial vowels were inserted at the same temporal location as the original ones. In a successive step, pitches and amplitudes of the original vowels were convolved to the semi-artificial ones, resulting in a syllable duration of 153 ms. This procedure is particularly fruitful in that it enables to produce semi-artificial CV syllables with fully controlled physical attributes, such as duration, pitch, timbre, and harmonics^67^.

### Phonetic discrimination training

In the time period between repeated EEG measurements (i.e., at T0 and T1, see the experimental procedure), the TG performed one hour of phonetic discrimination training consisting in judging whether pairs of CV syllables (i.e., a continuum between/gu/and/go/) were acoustically identical or not by pressing the corresponding mouse buttons (i.e., two alternatives forced-choice task with emphasis on accuracy and not on speed). Thereby, participants received visual trial-by-trial feedback (i.e., red or green circle presented in the middle of the screen) as well as cumulative feedback during each block (i.e., % correct and incorrect responses, red and green bars at the left and right side of the screen). The two original CV syllables/gu/(i.e., *f*0 = 130 Hz, F1 = 364 Hz, and F2 = 860 Hz) and/go/(i.e., *f*0 = 130 Hz, F1 = 480 Hz, and F2 = 860 Hz) were further manipulated by shifting F1 in steps of 4-Hz between 360–480 Hz, resulting in 30 acoustically different stimuli. All stimuli had a duration of 153 ms, the SOA of the syllable pairs was of 700 ms, and ITI corresponded to 1300 ms. During the training session, the stimuli were presented pairwise in a fully randomized order (i.e., 6 blocks of 10 minutes each). During every block each of the 30 stimuli was presented 8 times, resulting in 240 stimulus pairs per block. The first CV syllable of the pairs was always/gu/(i.e., *f*0 = 130 Hz, F1 = 364 Hz, and F2 = 860 Hz), whereas the second one was one out of the 30 variations.

### Discrimination test

In order to compare phonetic discrimination performance across the two groups (i.e., TG and PG) before and after training (i.e., training or passive exposure), all subjects performed a short phonetic discrimination test at T0 and T1 (i.e., before EEG at T0 and after EEG at T1) consisting of judging whether pairs of CV syllables are identical or not. Thereby, subjects heard exactly the same CV syllables (i.e., see next paragraph) that were presented during the training- (i.e., TG) and passive exposure (i.e., PG) sessions (i.e., continuum between/gu/and/go/, the first syllable was always/gu/). The solely difference is that here we used a reduced pool of stimuli (i.e., in the F1 range between 364–476 Hz, steps of 8 Hz, totally 15 acoustically different CV syllables, duration = 153 ms) that were presented in a randomized order (i.e., SOA = 700 ms, ITI = 1300 ms). Each stimulus was presented 4 times, resulting in a total of 60 trials. The phonetic discrimination test had a duration of about 2 minutes and was evaluated according to mean percent correct trials at T0 and T1.

### Experimental procedure

The volunteers were randomly assigned to two groups, namely to the TG or PG. Prior to the EEG session, all subjects performed the psychometric tests as well as pure tone audiometry in order to exclude any hearing problems. Afterwards, participants underwent the phonetic discrimination test (i.e., for quantifying phonetic discrimination at T0 and T1) and started with EEG measurements (i.e., T0, FFR followed by MMN). The stimuli were delivered via headphones (Sennheiser, CX-350, Colchester, Essex, UK) while watching a silent movie. Subsequently, the TG performed one hour of phonetic discrimination training, whereas the PG was passively exposed to the same stimulus material while watching a silent movie. At the end of the treatment, the two groups underwent the second EEG session (i.e., T1, FFR followed by MMN) and accomplished the second part of the phonetic discrimination test. The entire experiment lasted about four hours.

### EEG data acquisition

Continuous EEG (i.e., 32 electrodes +2 eye channels, provided by Easy Cap, forehead ground) was recorded with a sampling rate of 5 kHz and a high pass filter of 0.1 Hz by using an EEG-amplifier (Brainproducts, Munich, Germany). This specific device has previously been shown to reliably enable the collection of both cortical and subcortical brain responses^68^. The electrodes (i.e., sintered silver/silver-chloride) were located at frontal, temporal, parietal and occipital scalp sites according to the international 10–10 system. Data were collected by using linked earlobes- (i.e., FFR) or nose (i.e., MMN) references, and electrode impedance was reduced to <5 kΩ by using electrogel conductant. For all pre-processing steps, we used the Brain Vision Analyzer software package (Version 2.01, Brainproducts, Munich, Germany) and MATLAB (version 2013b). Stimulus presentation and the collection of behavioral responses were controlled by the “Presentation” software (Neurobehavioral Systems, Albany, California).

### FFR: data acquisition and processing

FFTs were evaluated in response to 3000 CV syllables (i.e.,/go/, *f*0 = 130 Hz, F1 = 480 Hz, F2 = 860 Hz, duration = 153 ms, SPL = 85 dB, SOA = 217 ms) of each polarity (i.e., a total of 6000 presentations). During EEG measurements, the audio waveform was recorded as an additional EEG channel, and triggers were recomputed offline by using thresholding functions in MATLAB (version 2013b). The data were filtered offline between 100–1000 Hz (i.e., butterworth filter, 48 dB/oct), and artefacts exceeding ± 50 µV were automatically rejected. Furthermore, responses were segmented into single sweeps of 173 ms (including a pre-stimulus baseline of 20 ms), baseline corrected, and averaged separately for each polarity. In order to bias higher-frequency components by maximizing the spectral response, waveforms to positive and negative polarities were subtracted^10^, and peak amplitudes of the harmonics were extracted for each participant by applying fast Fourier transformation (i.e., FFT). FFRs were computed over the steady-state portion of the response and for each participant spectral response amplitudes at electrode Cz were calculated over 1 Hz-wide bins surrounding *f*0 and harmonics. In addition, in order to demonstrate that the FFRs were not an artefact of electromagnetic interference induced by the headphones, we performed stimulus-response correlations (i.e., cross-correlation over all sample points) and expected a lag in the range of 6–10 ms^13^. Stimulus-response cross-correlations were also computed to evaluate training-related changes (i.e., training or passive exposure) in response fidelity to the stimulus periodicity (i.e., maximal correlation between the two signals). The collection of FFRs had a duration of about 21 minutes.

### MMN: data acquisition and processing

MMN responses were collected in order to test the hypothesis that short-term phonetic discrimination training may have an influence on the response properties of the auditory cortex. The same paradigm was also used to address a second more speculative research question, namely the putative influence of phonetic discrimination training on the processing of temporal speech information. Such a relationship was tackled based on previous work indicating a high degree of interaction between spectral and temporal parameters in the auditory-cortex^69^. Accordingly, we used a double-deviant MMN paradigm^70^ consisting of simultaneously varying F1 and duration of the deviants. Previous work has shown that double-deviant stimuli elicit a MMN with two peaks that correspond to the MMNs elicited by the two single deviations presented in isolation^70^.

The stimulus material consisted of 840 standards (i.e.,/gu/, duration = 153 ms, *f*0 = 130 Hz, F1 = 364 Hz, F2 = 860 Hz) and 120 deviants (i.e.,/go/, duration 153 ms, *f*0 = 130 Hz, F1 = 480 Hz, F2 = 860 Hz) which were presented with a presentation level of 70 dB SPL and a SOA of 700 ms. Stimuli were presented in a pseudorandomized order, whereby the deviant syllable was followed by at least one standard. EEG data were filtered offline between 1–30 Hz, and artefacts (i.e., eye movements and blinks) were eliminated by using an independent component analysis (i.e., ICA)^71^ in association with an automatic raw data inspection ( ± 100 µV). Afterwards, the data were segmented separately for standards and deviants into single sweeps of 700 ms, including a pre-stimulus baseline of 200 ms. The single sweeps were baseline corrected, and single-subject averages were computed separately for standards and deviants. MMN waveforms were calculated by subtracting AEPs in response to standards from those elicited by deviants. Based on grand average waveforms, single-subjects inspection, and topographies, MMN areas and latencies were extracted in the time range of 100–200 (i.e., MMN in response to spectral manipulation) and 200–300 (i.e., MMN in response to temporal manipulation) ms post stimulus onset at electrode FCz (i.e., showing maximal amplitudes). The MMN paradigm had a duration of about 11 minutes.

### MMN: Source estimation

In order to corroborate that MMN responses essentially originated from the auditory cortex^58^, intra-cortical maxima were evaluated by using a source estimation approach (i.e., LORETA, ref. 72). Thereby, we estimated the cortical origin of MMN responses separately for the two groups (i.e., TG and PG), the two time points (i.e., T0 and T1), and spectral and temporal manipulations in the time range between 100–200 (i.e., spectral manipulation) and 200–300 ms (i.e., temporal manipulation). This approach, unlike conventional dipole fitting, does not require a-priori assumptions about the number and the localization of the dipoles. LORETA calculates the three dimensional distribution of electrically active neuronal generators in the brain as a current density value (i.e., ∞µA/mm^2^), and provides a solution for the inverse problem by assuming that the smoothest of all possible activity distributions is the most plausible one for explaining the data. The characteristic feature of this particular inverse solution approach is the low spatial resolution which conserves the location of maximal activity but with a certain degree of dispersion^73^. Here, we determined the current density distribution for epochs of brain electrical activity on a dense grid of 2394 voxels at 7 mm spatial resolution. The localization error of LORETA’s source identification may vary between 7^72^ and 14 mm^74^. LORETA refers to a three-shell spherical model registered to the Talairach human brain atlas and source estimations are provided as x, y, z coordinates situated relative to the inter-commissural line (AC-PC line) in horizontal (x), the anterior/posterior (y), and vertical (z) directions. The solution space is confined to the grey matter portion of the human cortex, which rules out the option that subcortical tissue and white matter contribute to the solution.

### Statistical analyses

All statistical analyses of the EEG data (i.e., group comparisons and correlations, FFRs and MMN responses) were performed by using normalized percent signal change values, according to the following formula: % signal change = [(T1 value − T0 value)/T0 value] × 100. This procedure is particularly fruitful in that it enables to control for inter-individual variability of cortical^75^ and subcortical brain responses^34^. Psychometric- and electrophysiological data (i.e., FFR spectral f1 peak, MMN area, and MMN latency) were evaluated by using t-tests for independent samples (two-tailed), whereas the behavioral data of the discrimination test were evaluated according to a generalized linear mixed model for binomially distributed outcome with group (TG and PG) and time point (T0 and T1) as fixed factors, and participants as random factors. Furthermore, in order to exclude that FFRs were a simply artefact of electromagnetic interference induced by the headphones, the lag of the FFRs at T0 was tested against zero. Finally, within the TG putative relationships between cortical and subcortical responses as well as between subcortical signal change and learning performance (i.e., Δ percent correct responses between run 6 and run 1 of the training session) were assessed by using Pearson’s correlation. Based on the results clearly showing neural adaptation at the processing level of the brainstem after training, the relationship between training-related subcortical brain changes and learning improvement was tested in a one-tailed fashion. Otherwise, since we did not have clear a-priori hypotheses about the direction of cortical-subcortical coupling mechanisms, the correlation between auditory cortex and brainstem signal change was tested in a two-tailed manner. All omnibus tests as well as post-hoc analyses were corrected for multiple comparisons by using the Bonferroni procedure.
